# Pollution Impacts on Bacterioplankton Diversity in a Tropical Urban Coastal Lagoon System

**DOI:** 10.1371/journal.pone.0051175

**Published:** 2012-11-30

**Authors:** Gigliola R. B. Salloto, Alexander M. Cardoso, Felipe H. Coutinho, Leonardo H. Pinto, Ricardo P. Vieira, Catia Chaia, Joyce L. Lima, Rodolpho M. Albano, Orlando B. Martins, Maysa M. Clementino

**Affiliations:** 1 Laboratório de Microrganismos de Referência, Instituto Nacional de Controle de Qualidade em Saúde, Fundação Oswaldo Cruz, Rio de Janeiro, Brazil; 2 Diretoria de Programa, Instituto Nacional de Metrologia, Qualidade e Tecnologia, Rio de Janeiro, Brazil; 3 Instituto de Bioquímica Médica, Universidade Federal do Rio de Janeiro, Rio de Janeiro, Brazil; 4 Departamento de Bioquímica, Universidade do Estado do Rio de Janeiro, Rio de Janeiro, Brazil; University of Delaware, United States of America

## Abstract

Despite a great number of published studies addressing estuarine, freshwater and marine bacterial diversity, few have examined urban coastal lagoons in tropical habitats. There is an increasing interest in monitoring opportunistic pathogens as well as indigenous microbial community members in these water bodies by current molecular and microbiological approaches. In this work, bacterial isolates were obtained through selective plate dilution methods to evaluate antibiotic resistances. In addition, 16S rRNA gene libraries were prepared from environmental waters and mixed cultures grown in BHI medium inoculated with Jacarepaguá lagoon waters. Denaturing gradient gel electrophoresis (DGGE) analyses showed distinct community profiles between environmental communities from each studied site and their cultured counterparts. A total of 497 bacterial sequences were analyzed by MOTHUR, yielding 245 operational taxonomic units (OTUs) grouped at 97% similarity. CCA diagrams showcased how several environmental variables affect the distribution of 18 bacterial orders throughout the three distinct habitats. UniFrac metrics and Venn diagrams revealed that bacterial communities retrieved through each experimental approach were significantly different and that only one OTU, closely related to *Vibrio cholerae*, was shared between them. Potentially pathogenic bacteria were isolated from most sampled environments, fifty percent of which showed antibiotic resistance.

## Introduction

Urban coastal lagoons are impacted environments that are highly affected by mixing of sediments, seawater, and continental freshwater. These aquatic bodies offer aesthetic, economic and recreational value and also function as catchments for stormwater runoff. Furthermore, in tropical regions reminiscent natural mangrove habitats provide protection, food and breeding areas for different animal and plant species [1].

Coastal urban lagoons are subjected to anthropogenic impacts that generate high sedimentation rates and eutrophication [2]. Their maintenance as functional and healthy ecosystems is essential for our future welfare [3]. According to the World Health Organization (WHO), 4.0% of all deaths and 5.7% of the global disease burden are due to water related illnesses, stemming from poor water quality, hygiene and sanitation [4]. Pollution of water bodies with feces originating from different sources could lead to pathogen transmission and, therefore, to waterborne diseases [5].

Indicator bacteria, including total and fecal coliform and *Enterococci*, have been widely used as a monitoring tool for microbial contamination of water [6]. Although their presence provides information regarding health risks associated to gastrointestinal pathogens, this indicator system does not predict specific pathogens or sources of fecal contamination [7]. Fecal pollution in aquatic environments can lead to serious consequences when enteric pathogens are present. Access to both clean water and sanitation is not widespread in developing countries. According to WHO, more than 50% of water associated diseases are microbial intestinal infections, causing at least 11,399 deaths worldwide due to cholera [8].

As human populations increase, anthropogenic impacts affect lakes, rivers and coastal ecosystems mainly due to discharge of sewage and chemical compounds such as pesticides, hormones and antibiotics that are largely used in human and veterinary clinics [9]. The main source of antibiotics in aquatic environments comes from wastewater discharge, manure disposal and aquaculture [10]. Furthermore, their indiscriminate use has accelerated the spread of antibiotic resistance genes. In addition, antibiotics are often produced by environmental microorganisms that harbor intrinsic resistance genes [11]. Aquatic environments are sites of intense genetic exchange, in which originally susceptible bacteria may become resistant by acquisition of DNA encoding resistance traits through horizontal gene transfer. This process represents a threat to public health since opportunistic pathogens may acquire drug resistance through this mechanism [12].

**Figure 1:**
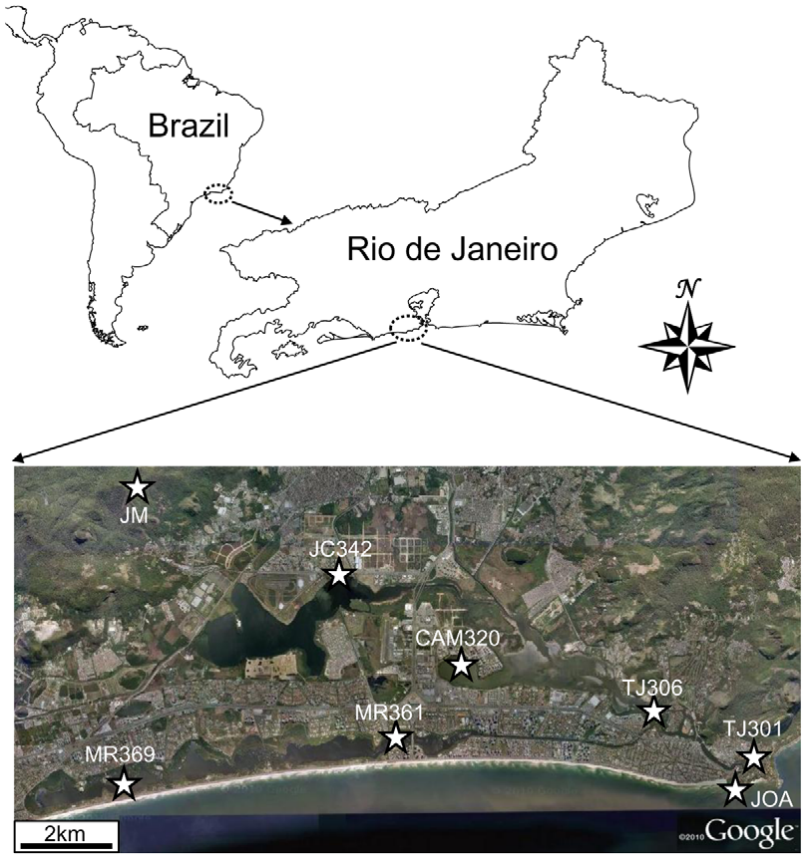
Sampling sites and experimental strategy. Stars (<$>\vskip -1 \scale 60%\raster="rg1"<$>) mark sampling points in the lagoon system.

Brazil holds around 15% of the Earth's freshwater reserve, distributed across more than 20,000 km^2^ of marshlands, rivers, lakes and estuaries. Water pollution occurs mainly in the most industrialized regions in the south and southeast that harbor nearly 60% of the Brazilian population [13]. Risks and consequences associated with microbial transmission in water bodies have been a concern in Brazilian public health issues since 1886 when Oswaldo Cruz earned his medical degree with a thesis addressing the transmission of waterborne microbes. Cruz demonstrated the occurrence of microbes in water from various sources based on physiological and chemical parameters and explored the general prevention of waterborne infections [14].

**Table 1:** Physico-chemical data from sampling stations.^1^

Sites	pH	Conductivity *	Turbidity **	O.D. ***	Salt (%)	Temp (°C)
JM	7.9	0.112	0	5.80	0.00	20.0
JC 342	7.1	4.8	139	5.52	0.25	24.3
CM 320	7.1	6.74	140	6.57	0.36	24.3
MR 369	7.1	9.88	74	4.97	0.55	24.2
TJ 306	7.1	17.8	45	5.50	1.05	24.0
MR 361	7.9	21.7	36	6.17	1.31	24.3
TJ 303	7.6	33.8	22	6.30	2.13	23.8
JOÁ	7.9	50.7	1	5.50	3.34	23.5

* *MiliSiemens* – mS/cm; ** Nefelométric Turbidity Unity – NTU; *** mg/L.

1 Additional information on physico-chemical parameters of the sampling sites can be found at INEA (http://www.inea.rj.gov.br).

Application of molecular methods has stimulated the interest in direct monitoring opportunistic and specific pathogens in surface waters [15]. One of the main procedures to evaluate microbial diversity, including pathogen occurrence, is the sequence analysis of environmental genes [16]. This approach can detect uncultured bacteria as well as conventional fecal indicators in water bodies, including *E. coli*, *Salmonella spp*. [17], *Shigella spp*. [18], *Campylobacter spp*. [19], *Legionellae* [20], and *Vibrio vulnificus* [21] among other known pathogens.

The aim of this study was to investigate how bacterial community diversity in a tropical coastal urban lagoon system is related to a pollution and salinity gradient going from terrestrial aquatic habitats up to the coastal Atlantic Ocean. A preserved freshwater environment from the Atlantic rain forest (JM) was used as a pristine control. The lagoon system, comprised of four major lakes (Jacarepaguá, Camorim, Tijuca and Marapendi), is located within the metropolitan area of Rio de Janeiro and covers approximately 280 Km^2^. The watershed of Jacarepaguá lagoon system is composed of several rivers that flow to lakes connected to the sea through Joá channel in Barra da Tijuca.

**Figure 2:**
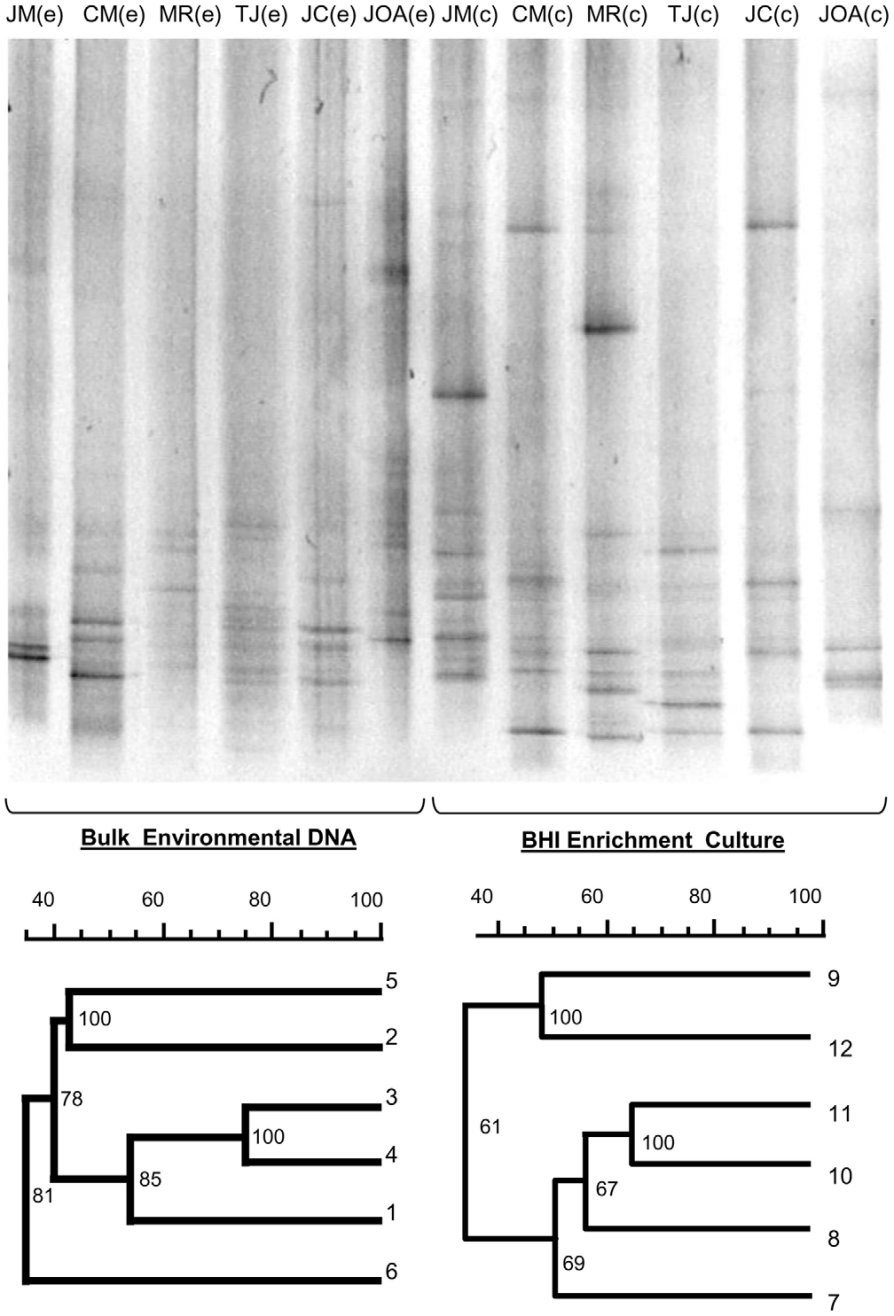
DGGE analysis of environmental and cultured samples. Dendogram generated using Bionumeric software based on images of DGGE band pattern profiles. Each sample is identified as environmental (e) or cultured (c).

## Materials and Methods

### Ethics statement

An ethics statement is not required for this work. No specific permits were required for the described field studies. The location is not privately-owned or protected in any way and did not involve endangered or protected species.

**Figure 3:**
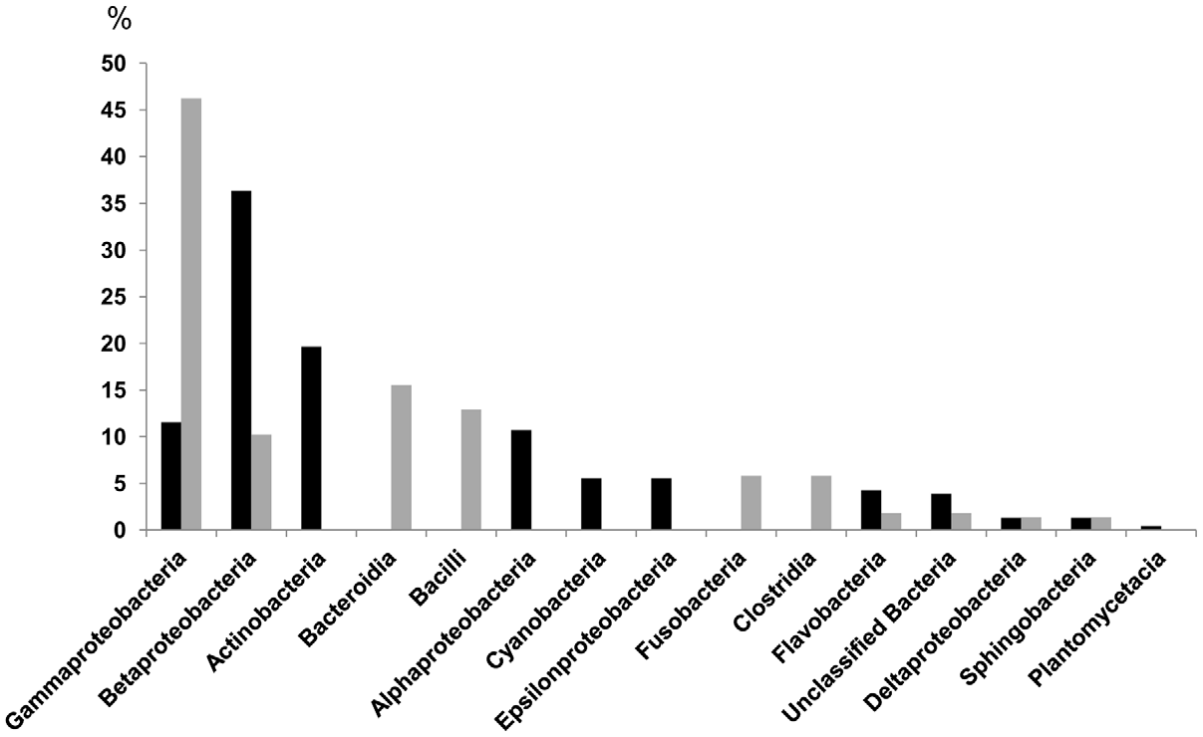
Bacterial taxonomic classes in environmental and cultured libraries. Sequences obtained from 16S rRNA gene libraries retrieved from environmental samples (black) and from enrichment cultures (grey) taxonomic assignment was performed through the RDP Classifier tool.

### Site description, experimental design and sampling

The coastal urban watershed of Jacarepaguá, located south of Rio de Janeiro (between latitudes 22°55′S and 23°03′S and longitudes 43°30′W and 43°18′W) is formed by rivers headwaters on the slopes of Tijuca and Pedra Branca massifs, with approximately 280 km^2^ of drainage area according to SMAC (Secretaria Municipal de Meio Ambiente). This basin is formed by Tijuca (4.34 km^2^), Camorim (0.80 km^2^), Jacarepaguá (4.07 km^2^) and Marapendi (3.33 km^2^) lakes that are connected to the Atlantic Ocean by Joá channel forming a tide influenced lagoon system. We selected eight sampling sites named: JM, JC342, CM320, MR361, MR369, TJ301, TJ306 and JOA (Figure 1). Samples at each station were obtained on April 16, 2009. JM station is located in a preserved site of the Atlantic rain forest (22°56′85′′S/043°24′6′′W) in a dam of a small pristine river that supplies freshwater to nearby communities. JC342 station (22°58′10′′S/43°22′99′′W) is located near the margin of Jacarepaguá lake close to Pavuna river. CM320 station (22°34′04′′S/043°55′32′′W) is in Camorim lake below the Ayrton Senna bridge. MR361 station (23°01′3′′S/43°25′26′′W) is in Marapendi lagoon at Chico Mendes park. MR369 station (23°00′17′′S/43°21′85′′W) is also in Marapendi lake near a biological reserve. TJ301 station (23°00′33′′S/43°17′62′′W) is in Tijuca lake near the connection to the sea. TJ306 station (23°00′59′′S/43°18′56′′W) is also in Tijuca lake near the junction between Camorin and Marapendi channels. JOA station (23°00′56′′S/43°17′60′′W) is in Joatinga channel under the Joá bridge. Samples of 5.8 L were collected in sterile polypropylene bottles at 1 m deep.

**Figure 4:**
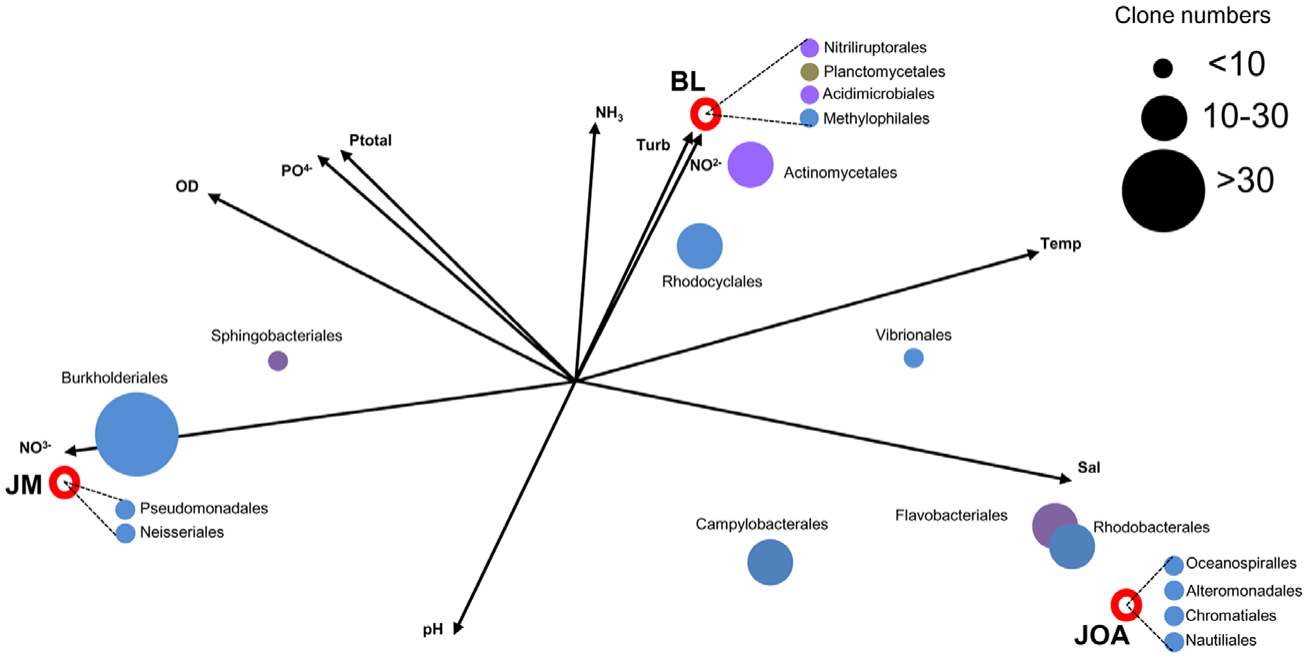
Environmental distribution of bacterial orders. Canonical correspondence analysis of sampling sites libraries from JM(e), BL(e) and JOA(e). Environmental parameters included in this analysis are: total phosphorus (Ptotal), ammonia (NH_3_), turbidity (Turb), nitrite (NO_2_^−^), temperature (Temp), salinity (Sal), pH, nitrate (NO_3_^−^), dissolved oxygen (OD) and orthophosphate (PO_4_^−^).

### Abiotic parameters

Water samples collected at the eight sites were analyzed for temperature, pH, conductivity, dissolved oxygen, turbidity and salinity at the time of sample collection using Water Quality Checker U-10 (HORIBA). Measurements of total phosphorus, orthophosphate, ammonia, nitrite and nitrate were provided by Instituto Estadual do Ambiente (INEA).

**Figure 5:**
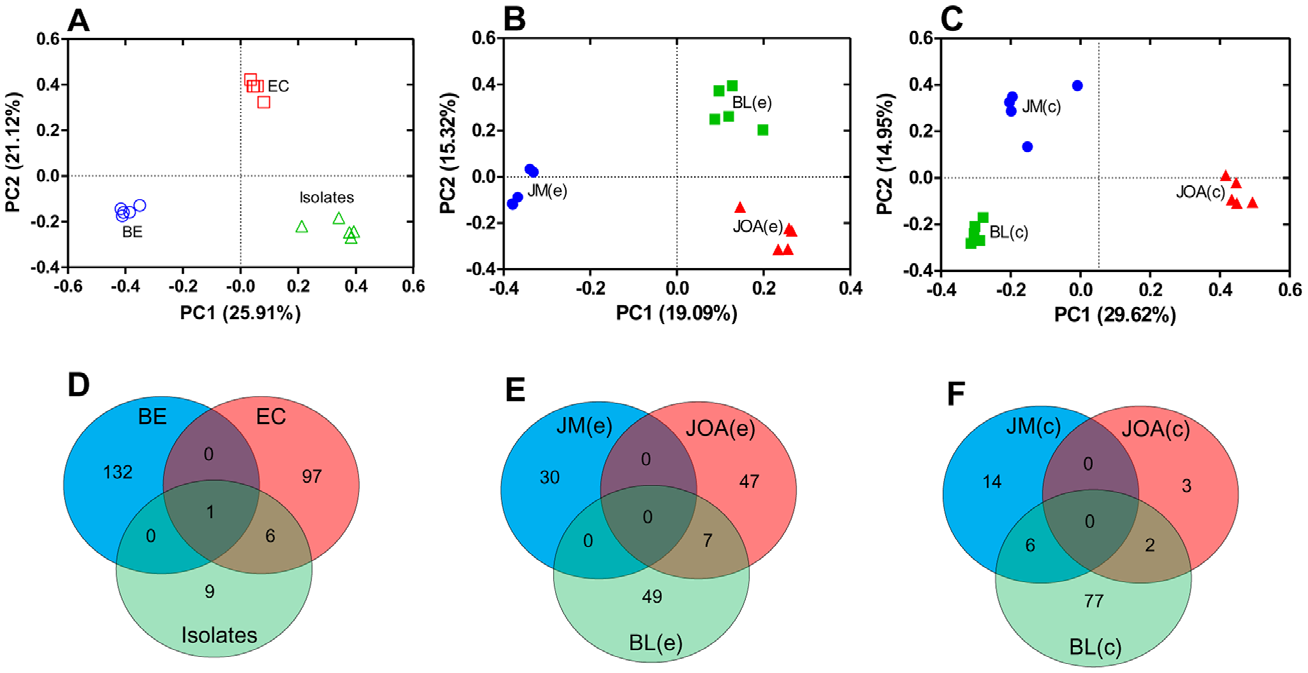
Match and similarities between bacterial communities. Principal coordinates plots (PCoA) were generated using the pairwise unweighted UniFrac distances. (**A**) Comparison between libraries from bulk-environmental DNA (BE), enrichment Cultures (EC) and isolates. (**B**) Comparison between bulk-environmental DNA libraries (e) of marine (JOA), brackish (BL) and freshwater (JM) sampling points, and (**C**) Comparison between enrichment culture libraries (c) of marine (JOA), brackish (BL) and freshwater (JM) sampling points. Venn diagrams (**D**) from libraries of bulk-environmental DNA (BE), enrichment cultures (EC) and isolates. (**E**) Venn diagrams from bulk-environmental DNA libraries (e) of marine (JOA), brackish (BL) and freshwater (JM) sampling points, and (**F**) Venn diagrams from enrichment culture libraries (c) of marine (JOA), brackish (BL) and freshwater (JM) sampling points.

### Bacterial isolation and enrichment

Isolation of bacterial strains was performed with inoculants retrieved from the eight sampling sites (Figure 1) using blood agar media (DIFCO) supplemented with 5% sheep blood and four types of commercially available selective culture media: Brain Heart Infusion (BHI) Broth and Agar (MERCK), Cetrimide Agar (CET) (DIFCO), MacConkey Agar (MAC) (MERCK) and Manitol Salt Agar (MSA) (MERCK). Media were prepared according to manufacturer's instructions and autoclaved for 20 min at 121°C. One milliliter of each sample was inoculated in 10 ml of BHI liquid media and incubated at 37°C. After 24 h, cultures were streaked onto BHI, CET, MAC, and MSA agar plates and then incubated at 37°C for 24 to 48 h. Representative morphotypes (twenty-two strains) were selected to perform taxonomic characterization by 16S rRNA gene sequencing. Pure cultures were preserved in glycerol 20% at −70°C.

**Figure 6:**
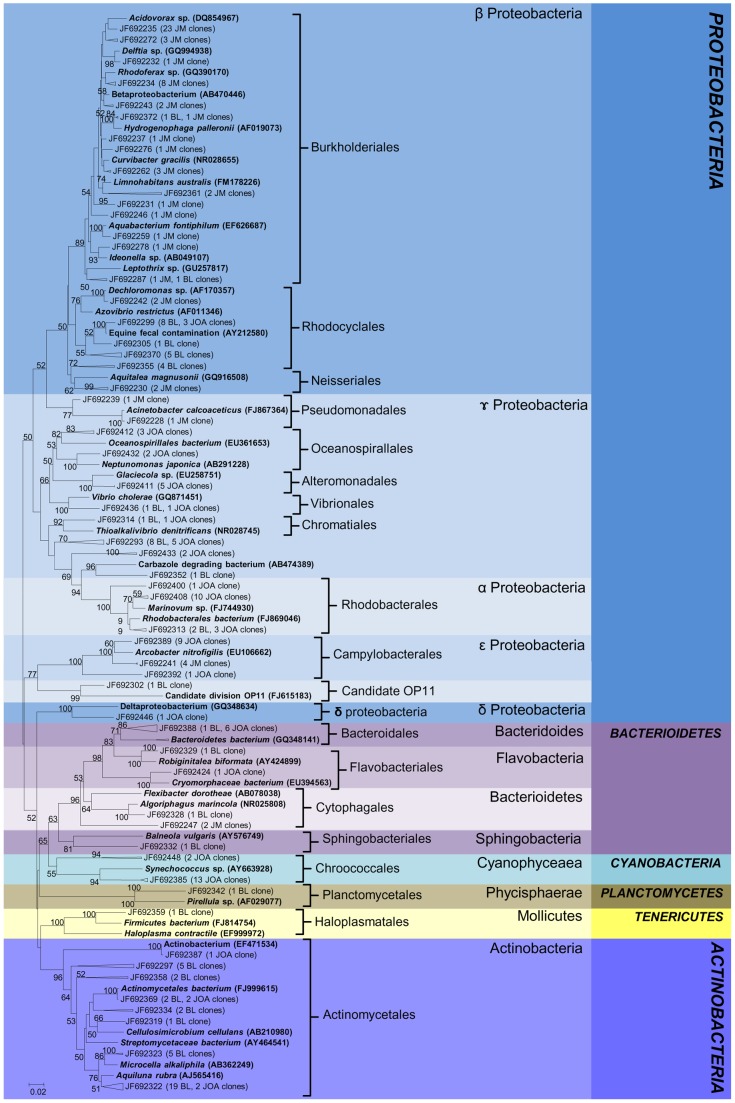
Phylogenetic tree of bacterial clones from environmental bulk DNA. Reference sequences from GenBank are shown in bold. OTUs were defined by using a distance level of 3% by the furthest neighbor algorithm in MOTHUR. Access number from each OTU is displayed. Tree topology is based on neighbor joining and bootstrap analysis was performed with 1000 replications. Bootstrap value <50 are not shown.

**Figure 7:**
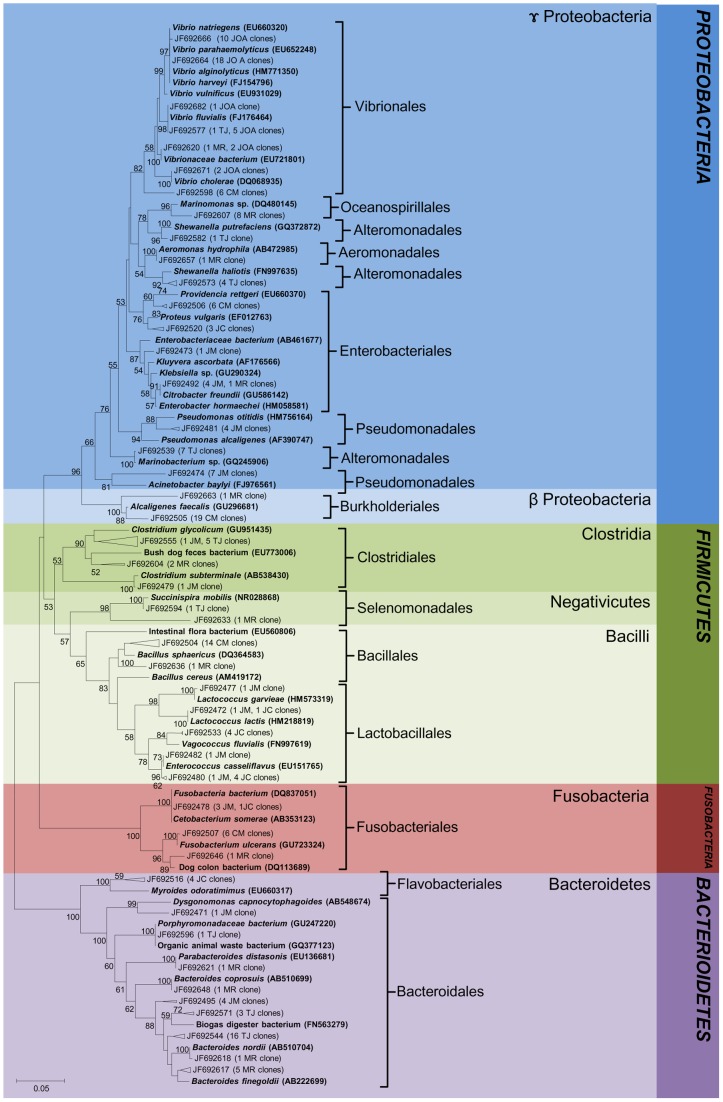
Phylogenetic tree of bacterial clones from BHI enriched culture. Reference sequences from GenBank showcased in bold. OTUs were defined by using a distance level of 3% by using the furthest neighbor algorithm in MOTHUR. Access number from each OTU is displayed. Tree topology is based on neighbor joining and bootstrap analysis was performed with 1000 replications. Bootstrap value <50 are not shown.

**Figure 8:**
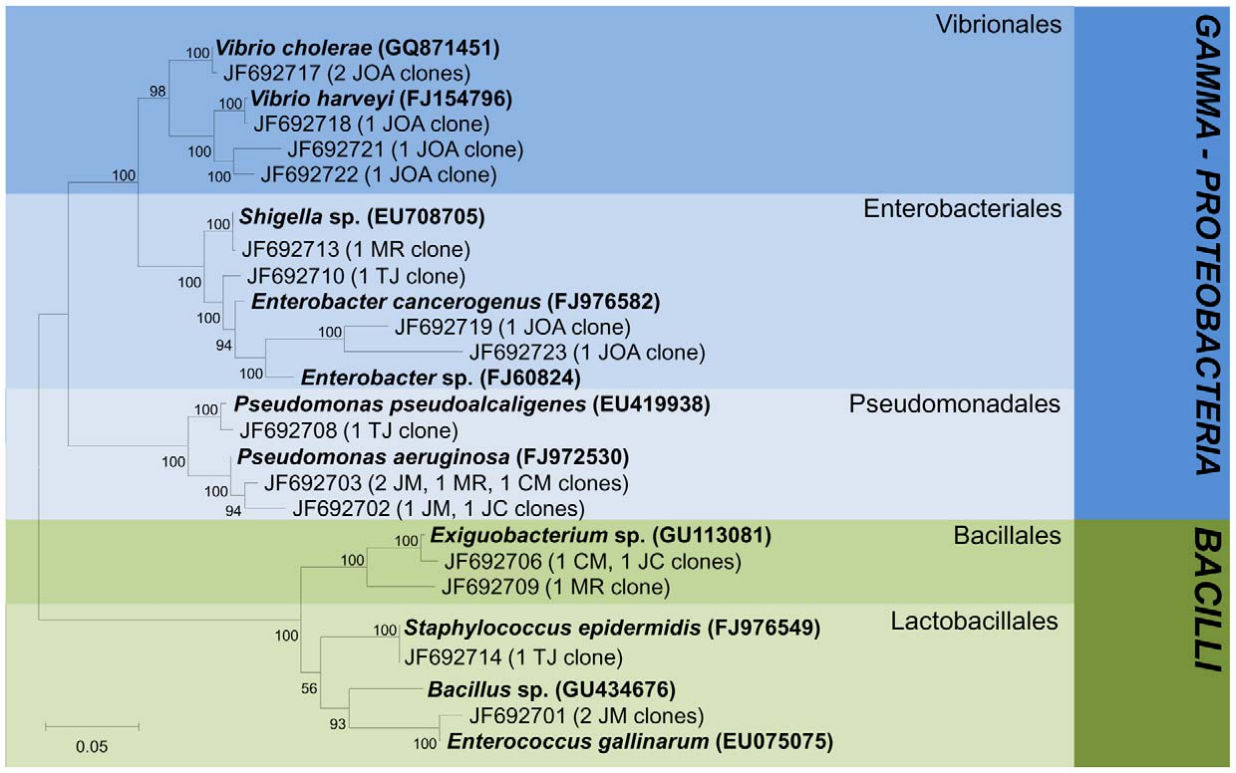
Phylogenetic tree of isolated bacteria. Reference sequences from GenBank are showed in bold. OTUs were defined by using a distance level of 3% by the furthest neighbor algorithm in MOTHUR. Access number from each OTU is displayed. Tree topology is based on neighbor joining and bootstrap analysis was performed with 1000 replications. Bootstrap values <50 are not shown.

Water samples were inoculated into Brain Heart Infusion (BHI), a rich culture medium, to promote growth of aerobic and facultative anaerobic bacteria. A one ml aliquot from each of the eight sampling sites (JM, JC342, CM320, MR361, MR369, TJ301, TJ306, and JOA – Figure 1), was inoculated in 10 ml of BHI and incubated at 37°C. After 24 hours, cultures were centrifuged and genomic DNA was extracted from microbial pellets using Qiagen DNeasy Blood & Tissue Kit following manufacturer's recommendations [22].

**Table 2:** Antibiotic resistance profile of isolated bacteria.

Isolate	NCBI (BEST HITS) Closest relative and similarity (%)	ACESSION NUMBER	SITE	Ceftazidime	ANTIBIOTIC Aztreonam	Tobramycin
**1**	1 *Enterococcus gallinarum (93%)*	EU075075	**JM**	R	R	R
**2**	2 *Pseudomonas pseudoalcaligenes (96%)*	EU419938	**TJ303**	S	R	S
**3**	2 *Exiguobacterium sp. (96%)*	GU120647	**TJ306**	R	R	S
**4**	2 *Exiguobacterium sp.(93%)*	HQ622549	**CM320**	S	R	S
**5**	2 *Pseudomonas aeruginosa (95%)*	HM461147	**MR361**	R	R	R
**6**	2 *Shigella sp.(96%)*	GU968176	**MR369**	S	S	R
**7**	2 *Exiguobacterium sp.(95%)*	AY745848	**JC342**	R	R	S
**8**	1 *Vibrio fluvialis (94%)*	FR695475	**JOA**	R	R	R
**9**	3 *Vibrio cholerae (96%)*	GQ871451.1	**JOA**	S	R	S
**10**	3 *Vibrio harveyi (95%)*	FJ154796.1	**JOA**	S	R	R
**11**	4 *Vibrio cholerae (98%)*	CP002555.1	**JOA**	R	R	S

1 Salt Manitol Agar.

2 Cetrimide Agar.

3 Blood Agar.

4 Mac Conkey Agar.

R, resistant and S, susceptive to antibiotic.

### Antimicrobial susceptibility testing

Determination of antibiotic resistance was performed by a disc diffusion method according to Clinical and Laboratory Standards Institute [23] interpretive criteria recommendations. Bacterial suspensions were adjusted to 0.5 McFarland standards and inoculated into Mueller-Hinton agar medium. Twenty antimicrobial discs were used as follows: ticarcillin/clavulanic acid 75/10 µg, piperacillin/tazobactam 100/10 µg, cefepime 30 µg, ceftazidime 30 µg, aztreonam 30 µg, meropenem 10 µg, imipenem 10 µg, ciprofloxacin 5 µg, gentamicin 10 µg, polymyxin B 300 IU, tobramycin 10 µg, norfloxacin 10 µg, tetracycline 30 µg, ampicillin 10 µg, cotrimoxazole 25 µg, sulfonamides 300 µg, doxycycline 30 µg, clarithromycin 15 µg, chloramphenicol 30 µg, ampicillin/sulbactam 10/10 µg.

### DNA extraction and 16S rRNA gene library construction

Water samples (5.8 L) were filtered through 0.2 µm Sterivex filters (Millipore) after filtration through a 3.0 µm filter to separate free-living microbes from larger organisms and particles. Total nucleic acids were isolated by cell lysis with proteinase K and SDS, followed by phenol-chloroform extraction as previously described [24]. DNA integrity was checked on a 1% (w/v) agarose gel. PCR was performed in 50 µl reaction mixtures (2.5 mM MgCl_2_, 0.2 mM deoxynucleoside triphosphates, 1 ng.µl^−1^ of each primer, 2.5 U of High Fidelity Platinum *Taq* DNA polymerase (Invitrigen), 1× PCR buffer and 200 ng of each environmental DNA sample, with the universal bacterial primers 27BF (5′-AGAGTTTGATCCTGGCTCAG-3′) [25] and 907RAB (5′-TTTGAGTTTMCTTAACTGCC-3′) [26]. PCR amplification was carried out with a 5 min denaturing step at 94°C; followed by 30 cycles at 94°C for 90 seconds, 50°C for 90 seconds, and 72°C for 2 min. The final step was an extension at 72°C for 5 min. PCR products were concentrated and purified with a GFx PCR DNA and Gel Band Purification Kit (GE Healthcare) after electrophoresis on a 1% (w/v) agarose gel. PCR products were cloned into the pGEM-T cloning vector (Promega) and used to transform competent *E. coli* DH10B cells. Positive colonies were picked and frozen in glycerol 20% at −70°C. While three environmental (e) 16S rRNA gene libraries were constructed from JM(e) (pristine freshwater), BL(e) (DNA pooled from six lake samples) and JOA(e) (coastal marine sample), eight BHI enrichment culture (c) libraries were constructed from JM(c), JC342(c), CM320(c), MR361(c), MR369(c), TJ301(c), TJ306(c) and JOA(c).

### DGGE analysis

Bacterial community diversity was analyzed by denaturing gradient gel electrophoresis (DGGE). The 16S rRNA gene fragments were PCR amplified using the specific primers 968BfC (5′-CGCCCGGGGCGCGCC CCGGGCGGGCGGAACGCGAAGAACCTTAC-3′) and 1401Br (5′-CGGTGTG TACAAGGCCCGGGAACG-3′) [27]. Amplicons (50 μl) from each sample were applied to the DCode DGGE system (BioRad) and ran at 60 V and 60°C for 18 h in 1× TAE buffer. The 6% (w/v) polyacrylamide gels were prepared with a denaturing gradient ranging from 45 to 60%. After electrophoresis, gels were stained with SYBR green I (Molecular Probes) for 1 h and then scanned using the Image Quant 300 (GE Healthcare) gel documentation imager.

### Sequence analysis and identification

Approximately 96 clones from each library were selected for sequence analysis. Plasmid DNA from each clone (400 ng) was prepared and sequences were obtained by cycle sequencing with the Big Dye Terminator v3.1 Cycle Sequencing Kit (Applied Biosystems) and analyzed in an Applied Biosystems ABI Prism 3730 automated DNA sequencer. Chromatograms were converted to Fasta format using Phred software and sequences with less than 300 bp were removed. A total of 494 valid sequences with Phred score ≥20 were compared with sequences from the Ribosomal Database Project II [28]. Sequences were also analyzed by BLAST searches in GenBank database and were aligned with representative bacterial sequences obtained from public databases using ClustalX software [29]. Partial 16S rRNA gene sequences generated in this study were deposited in GenBank under accession numbers JF692227-JF692723.

### Canonical Correspondence Analysis

To correlate bacterial diversity with environmental physical and chemical parameters of our sampling sites we performed a Canonical Correspondence Analysis (CCA) carried out through the software MVSP (*MultiVariate Statistical Package 3.1*, Kovach Computing Services). Partial 16S rDNA sequences retrieved from the eight sampling sites were distributed in 18 orders according to their taxonomic assignment performed through the RDP classifier tool. For practical purposes, sequences from six sampling sites located in the lagoon system (JC342, CM320, MR361, MR369, TJ301, and TJ306) were grouped as a single dataset named BL.

### Biodiversity and phylogenetic analyses

Chimeric sequences were removed by MOTHUR software [30]. Sequences were clustered as operational taxonomic units (OTUs) at 97% similarity. OTU diversity was examined using rarefaction analysis by the same software. Venn diagrams were also generated using MOTHUR. Phylogenetic trees were constructed from libraries using reference sequences obtained at GenBank by neighbor-joining algorithm, based on distances calculated by the Kimura-2 method. Phylogenetic analysis was performed with MEGA4 software [31] and bootstrap analysis was carried out with 1000 replications. Tree topology and hit distribution were uploaded to the UniFrac computational platform [32]. UniFrac analysis is a beta diversity metric that quantifies sequence similarities based on phylogenetic relatedness. In order to visualize the distribution of bacterial communities we used UniFrac to perform PCoA highlighted by significance. Libraries were randomly sub-sampled to test result consistency.

## Results

### Water chemistry and experimental design

Abiotic parameters of water samples collected at eight stations (Figure 1) were analyzed. Jacarepaguá and Camorin lakes had high levels of turbidity and low salinity, while low turbidity and high salinity were observed in Marapendi and Tijuca lakes (Table 1). To characterize bacterial communities present in these habitats, culture dependent and independent 16S rRNA gene libraries were constructed.

### DGGE analysis of bacterial communities

DGGE profiles retrieved from environmental DNA (BE) and enrichment cultures (EC) were markedly distinct, as well as within the six sites (JM, CM, MR, TJ, JC, JOA) (Figure 2). Relationships among communities resulted in a total of 12 different profiles. Environmental DNA banding patterns produced three clusters. The first included JC and CM lakes grouped at 40% similarity with the second cluster which contained three samples, two from the brackish water lakes MR and TJ, and the other from the freshwater site JM. The most distantly related community is from sea water (JOA) sharing 32% similarity with clusters 1 and 2. Profiles from the enrichment samples resulted in two main clusters related at 35% similarity. The first contained high saline stations MR and JOA (48%) while the second encompassed brackish (JC, TJ, CM) and freshwater (JM) lake samples.

### Taxonomic composition of 16S rRNA libraries

Using the RDP classifier tool, 16S rRNA gene sequences retrieved from our libraries were assigned to distinct taxonomic classes (Figure 3). Fourteen classes were identified: four of them (*Bacteroidia, Bacilli, Fusobacteria* and *Clostridia*) were exclusively present in enrichment culture (EC) libraries while five classes (*Actinobacteria, Alphaproteobacteria, Cyanobacteria, Epsilonbacteria* and *Planctomycetacia*) occurred only in environmental libraries (BE). *Gammaproteobacteria* was the most abundant class in EC while *Betaproteobacteria* predominated in BE.

The RDP classifier tool detected 18 bacterial orders between environmental sequences which were distributed in three different environments represented in a CCA diagram (Figure 4) as follows: *Actinomycetales*, *Flavobacteriales*, *Rhodobacterales* and *Vibrionales* were distributed between JOA and BL. *Burkholderiales* and *Sphingobacterales* were common to JM and BL. *Campylobacterales* was found in JOA and JM while *Rhodocyclales* was common to all three environments. All other orders were exclusive from one of the three sampling sites. Orders that include halotolerant organisms were detected exclusively in environments with higher levels of salinity (e.g. *Oceanospirillales* in JOA station and *Vibrionales* in JOA and BL). JOA and JM were positioned in extreme opposites of the CCA diagram; BL was positioned between them but closer to JOA suggesting these two sites share more ecological similarities between themselves than with JM.

### Bacterial diversity

Nine 16S rRNA gene libraries were analyzed, three environmental and six from enrichment cultures, yielding a total of 494 valid sequences. Environmental DNA obtained from the four brackish water lakes CM(e), MR(e), TJ(e) and JC(e) were pooled to build a single free living bacterial library henceforth named BL(e). Environmental libraries produced 243 sequences obtained from pristine freshwater JM(e), brackish lagoon water BL(e) and coastal marine seawater JOA(e). A total of 229 sequences were obtained from the six enrichment culture libraries JM(c), JC(c), MR(c), TJ(c), CM(c) and JOA(c). The remaining 22 sequences were recovered from Jacarepaguá lagoon bacterial isolates.

Unweighted UniFrac was used to cluster bacterial 16S rRNA gene sequences according to shared similarities in community composition and applied to simultaneously compare three different data sets through principal coordinate analysis (PCoA) (Figure 5). Initially, sequences from environmental libraries JM(e), BL(e) and JOA(e) were combined as a single data set (BE) and compared with sequences from enrichment cultures (EC) and bacterial isolates (Figure 5A). In the scatter plot, bacterial clusters retrieved by the three different methodological approaches were significantly different. Libraries from environmental sequences (BE) and cultured bacteria (EC and isolates) were separated by the first principal component. Next, environmental pristine freshwater JM(e), Jacarepaguá lagoons BL(e) and coastal seawater JOA(e) libraries were analyzed by PCoA (Figure 5B). The first principal component separated seawater and brackish water libraries from the freshwater sample JM(e). Regarding cultured communities, when JM(c) was compared with BL(c) and JOA(c), principal component 2 separated libraries by the salinity of their original environment (Figure 5C). Community similarity was also assessed through Venn diagrams using OTUs grouped in a similarity level of 97%. First, OTUs from the three methodological approaches (BE, EC and isolates) were compared (Figure 5D). A single OTU is shared between these three datasets, while six were shared between EC and isolates and none were shared between BE and EC or BE and isolates. When analyzing exclusively sequences from environmental samples only JOA(e) and BL(e) shared OTUs (Figure 5E). While sequences from cultures BL(c) shared six OTUs with JM(c) and 2 with JOA(c), none were shared between JM(c) and JOA(c) or between the three datasets (Figure 5F).

### Phylogenetic analysis

The identity of each 16S rRNA sequence was determined by BLAST-n searches against the NCBI GenBank database. Bacterial sequences from JM(e) were dominated by *Betaproteobacteria*, especially by members of *Burkholderiales*, *Rhodocyclales* and *Neisseriales* (Figure 6). Two *Cytophagales* clones, four *Arcobacter nitrofigilis* and one clone related to *Acinetobacter* were observed in the freshwater environment. In brackish waters BL(e), the most abundant groups were *Actinomycetales* followed by *Rhodociclales and Rhodobacteriales* that shared the same OTUs with seawater JOA(e). *Vibrionales, Chromatiales, Bacteroidales, Flavobacteriales, Sphingobacteriales* and *Planctomycetales* were observed in a smaller proportion. Nine clones related to bacteria originating from equine fecal contamination and a single clone related to candidate division OP11 were also observed in BL(e). In JOA(e), *Cyanobacteria* represented by *Chroococcales* and *Gammaproteobacteria* were dominant groups while only a few sequences were related to *Actinobacteria, Deltaproteobacteria* and *Bacteroidetes*. We also observed two clones affiliated to *Vibrio cholerae*, one in seawater and another in the lagoon environment. Sequences obtained from enrichment cultures with different inoculants were distributed into four main bacterial phyla: *Proteobacteria, Firmicutes, Bacteroidetes and Fusobacteria* (Figure 7). In JM(c), clones related to *Pseudomonadales* were the most abundant, followed by those related to *Bacteroidales, Enterobacteriales, Lactobacillales, Fusobacteriales and Clostridiales. Enterobacteriales* was retrieved in brackish water from lakes CM(c), MR(c) and JC(c) while *Pseudomonadales* was present in TJ(c) lake. Clones related to *Clostridiales and Bacteroidales* were shared between MR(c) and TJ(c) lakes while sequences related to *Lactobacillales* were detected in JC(c) lake. Sequences related to *Fusobacteriales* were observed in CM(c) and MR(c) and one clone in JC(c) samples. In MR(c), sequences related to *Oceanospirallales* and *Aeromonadales* were detected. However, clones related to *Burkholderiales* and *Bacillales* were shared with CM(c) and *Selenomonadales* with TJ(c) environments. *Alteromonadales* were observed only in lake TJ(c). The *Vibrionales*, widely represented by several clones, were found in marine water JOA(c) and in lake samples CM(c), TJ(c) and MR(c). We also analyzed phylogenetic relationships of bacteria isolated from freshwater JM(i), brackish water from Jacarepaguá JC(i), TJ(i), MR(i) and CM(i) and marine seawater JOA(i) (Figure 8). Bacterial isolates were composed of two phyla, *Proteobacteria* (72%) and *Firmicutes* (28%) with members divided into *Lactobacillales, Enterobacteriales, Vibrionales, Bacillales* and *Pseudomonadales* orders. Bacteria isolated from JM(i) belonged to *Enterococcus gallinarum* and *Pseudomonas aeruginosa*, which were also isolated from polluted MR(i), CM(i) and JC(i) lakes. Isolates recovered from CM(i), JC(i), MR(i) and TJ(i) samples were related to *Shigella* sp., *Pseudomonas pseudoalcaligenes*, *Staphylococcus epidermidis*, and *Exiguobacteriam* sp.. Bacterial isolates from seawater JOA(i) were affiliated to *Vibrionales* and *Enterobacteriales*. A single OTU, closely related to *Vibrio cholerae*, was shared between the three methodological approaches employed in this study.

### Antibiotic susceptibility

From a total of 22 representative morphotypes obtained from different sites at lagoon ecosystems, 11 isolates demonstrated resistance to at least one antibiotic, showcasing five distinct profiles of resistance to β-lactams and aminoglicosides (Table 2). Among twenty antimicrobial test-discs, aztreonam presented the highest resistance profile (91%) as previously demonstrated in the *P. aeruginosa* strains isolated from treated hospital wastewater released in that same aquatic ecosystem [33]. Sequences from the Jacarepaguá bacterial collection were affiliated with members of *Lactobacillales, Enterobacteriales, Vibrionales, Bacillales* and *Pseudomonadales* orders (Figure 8). Simultaneous resistance to three antibiotics was observed in *Enterococcus gallinarum* from JM, *Pseudomonas aeruginosa* from MR361 and *Vibrio fluvialis* from JOA. In addition, two antibiotic resistant *Vibrio cholerae* isolates from JOA station were detected.

## Discussion

Jacarepaguá lagoon ecosystem is a dynamic environment due to mixing of sediments, seawater and continental freshwater impacted by metropolitan pollution. Shifts in physical, chemical and microbiological properties in lagoon and adjacent coastal marine environments can occur in short time periods, driven by marine tides, rainwater runoff and sewage flow, creating an intense selective pressure that influences bacterioplankton community composition in these urban brackish bodies [24], [34]. Comparative environmental analysis by CCA (Figure 4) and phylogenetic community comparison by PCoA (Figure 5B) suggest that costal seawater (JOA) and brackish lagoons (BL) develop ecologically similar bacterial communities, probably due to water mixing resulting from tidal variation. Atlantic rain forest freshwater (JM) resulted in lower similarity with the two other sites. Bacterial phylogenetic diversity in these environments is mainly structured by salinity, which explains the distribution of three sampling sites in the CCA diagram: seawater (JOA) and freshwater (JM) are clearly separated and the brackish environment (BL) occupies an intermediate position between them. UniFrac metrics showed that bacterial diversity retrieved from uncultured communities and enrichment cultures were significantly different. PCoA data showed a clear separation between environmental and cultured communities from freshwater (JM), brackish water (BL) and seawater (JOA) samples, corroborating that salinity, rather than temperature and pH, may defines microbial community composition and biogeography as described in other systems [32], [35].

It was easy to ascertain through Venn diagrams that a very small number of OTUs were shared between libraries generated from DNA obtained by culture dependent (enrichment and bacterial isolation) and independent methods, suggesting that different bacterial communities are retrieved by each methodology. *Vibrio cholerae* occurrence was detected in seawater and in brackish lagoon environments by all methodological approaches used in this study. Aquatic bacteria might be indigenous to environments or occasionally present in water shedding from human, animal, plant or soil materials [36]. The phylogenetic tree showed significant diversity originating from fecal and pathogenic bacteria residing in polluted lagoon environments (Figure 6).

Fifty percent of the isolates from Jacarepaguá collection were antibiotic resistant bacteria, including species that are known human pathogens such as *Pseudomonas aeruginosa* and *Vibrio cholerae*. Among lagoon isolates, *Enterococcus gallinarum, P. aeruginosa* and *Vibrio fluvialis* showed multi resistance (Table 2). The high level of aztreonam resistance among isolates suggests that environmental strains may act as a reservoir of resistance genes in aquatic ecosystems [37]–[38]. This achievement may be linked to the occurrence of extended spectrum beta-lactamases (ESBL) and/or ampC-producing organisms, capable of hydrolyzing a large spectrum of cephalosporins, penicillins, and aztreonam [39]. The presence of pathogenic bacteria in polluted lagoons is expected but when these microbes carry genes that confer resistance to conventional drugs these environments pose serious risks to public health [40]. Performing antibiotic susceptibility tests on isolated bacteria is a way to access risks to humans and animals in public environments associated with impacted water bodies. Although numerous previous studies have demonstrated extensive antibiotic resistance in clinical human isolates, much of the immense reservoir of prevalent antibiotic resistance genes present in environmental settings has not been fully described [12], [41]–[42].

Currently, a complementary technique for searching antibiotic resistant genes is metagenomic functional selection, which has demonstrated that the repertoire of resistance genes is much more diverse than previously suggested through the use of culture-dependent methods [43]–[44]. Alternatively, DNA probes for specific drug resistance genes can be used in metagenomic surveys. Regulatory agencies continue to use traditional indicator counts as a measure of health risk; especially at the local level there is no move toward a sequence based approach to this issue given both costs, technical skill needed and the fact that there is no health assessment available for such sequence analyses. However, massively paralleled deep sequencing approaches are indeed growing in use for community analysis and in the not-so-distant future novel genes and indicators to assess water quality will be revealed.
